# Supplementary figures and images for: Prevalence and spectrum of germline BRCA1 and BRCA2 mutations in multiethnic cohort of breast cancer patients in Brunei Darussalam
Source: PLoS One. 2025 Jun 18;20(6):e0312635. doi: 10.1371/journal.pone.0312635 (PMC12176220; doi:10.1371/journal.pone.0312635)

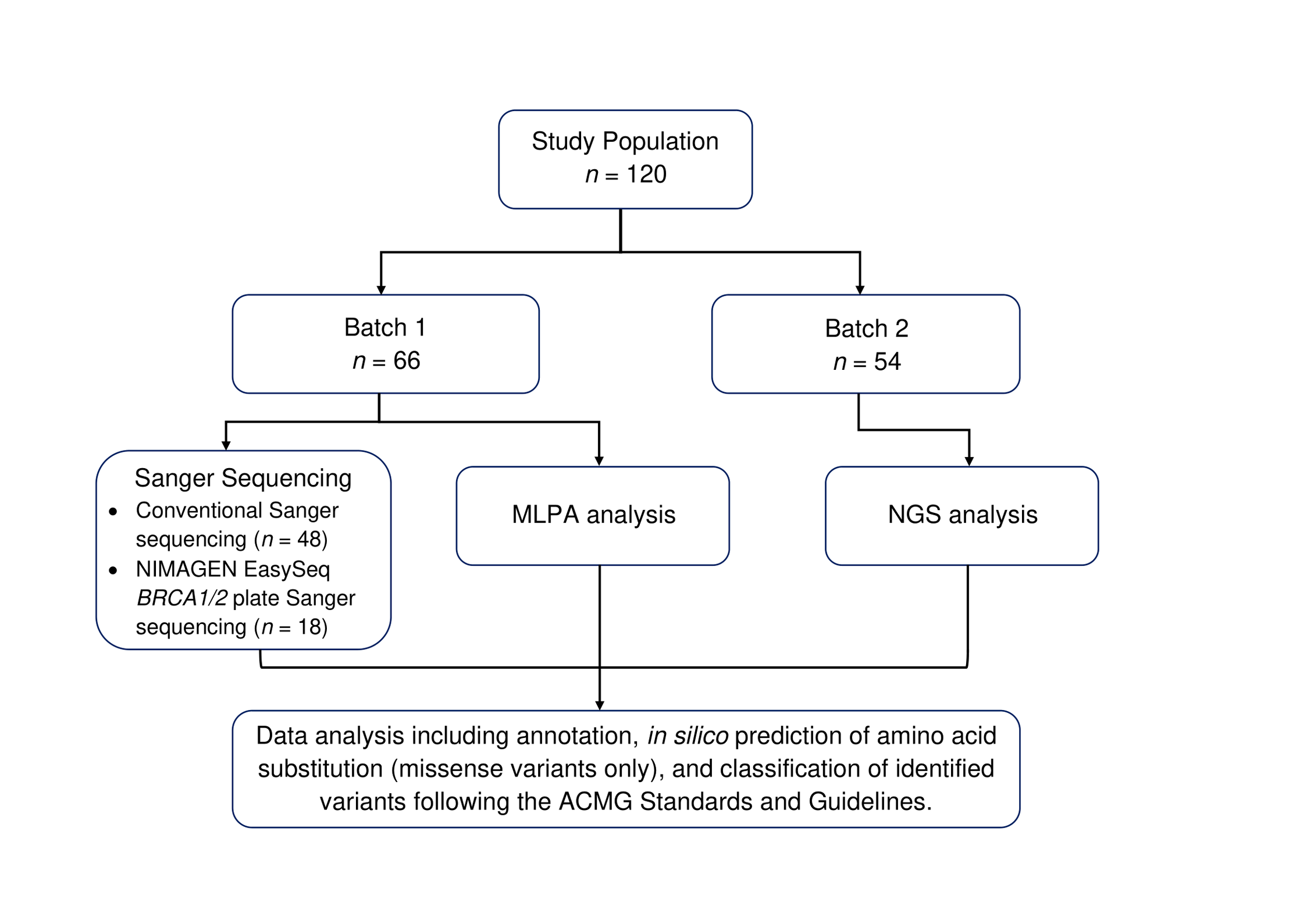

Supplement: S1 Fig — (TIF) [file pone.0312635.s001.tif]

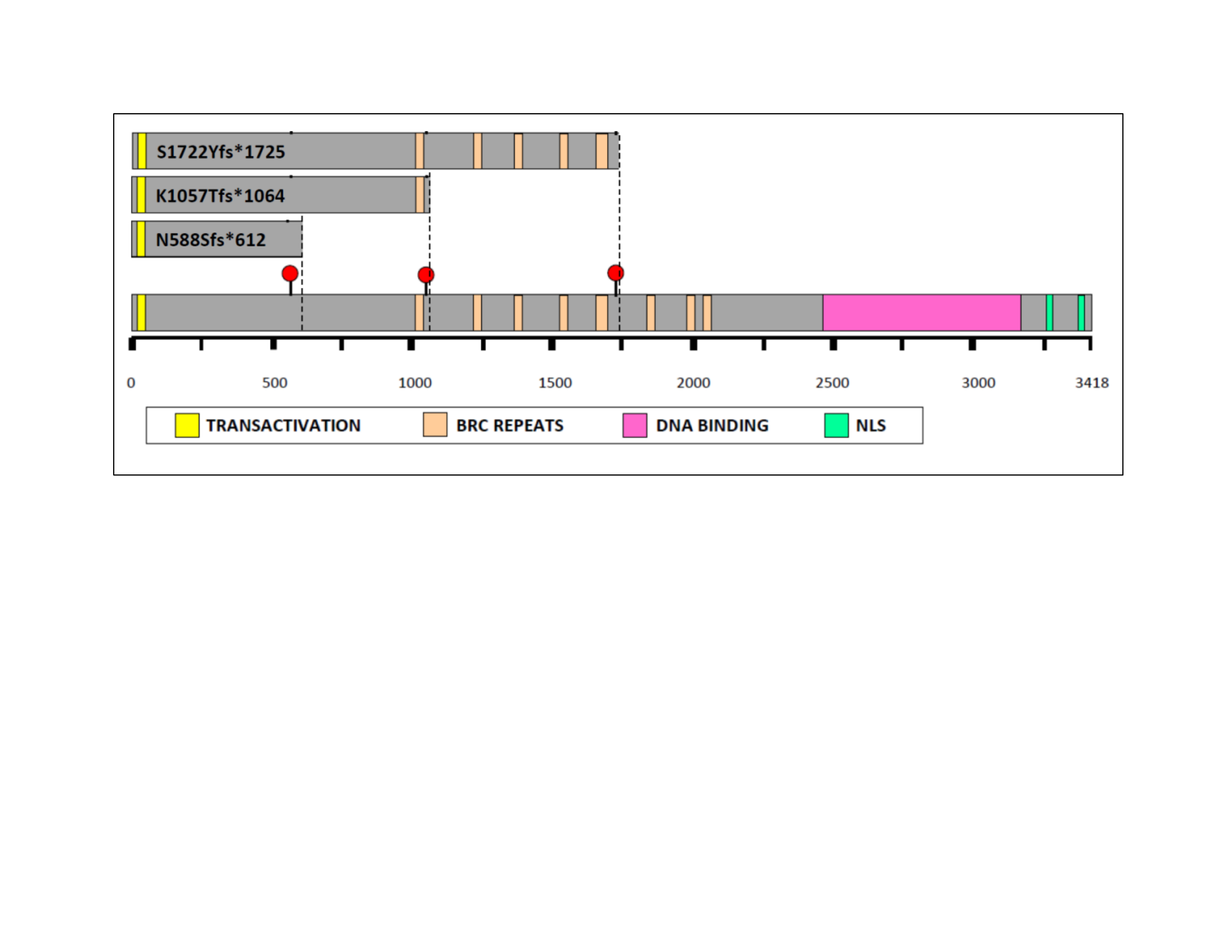

Supplement: S2 Fig — The three identified BRCA2 pathogenic mutations, c.1763_1766delATAAA, c.3170_3174delAGAAA and c.5164_5165delAG led to BRCA2 protein truncation at amino acids positions 612, 1064, and 1725 respectively. The truncation caused the loss of important domains affecting the function of BRCA2 predominantly in the HR-regulated DNA repair pathway. NLS; Nuclear localization signals. (TIF) [file pone.0312635.s002.tif]
